# Occurrence and Multi-Locus Genotyping of *Giardia duodenalis* in Black Goats from Fujian Province, China

**DOI:** 10.3390/ani15020199

**Published:** 2025-01-13

**Authors:** Shou-Xiao Huang, Kai Hu, Peng-Fei Fu, Si-Ang Li, Yang Liu, Zhipeng Niu, Dong-Hui Zhou

**Affiliations:** 1 Key Laboratory of Fujian-Taiwan Animal Pathogen Biology, College of Animal Sciences, Fujian Agriculture and Forestry University, Fuzhou 350002, China; huabgsx@hotmail.com (S.-X.H.); hukai86@163.com (K.H.); fupengfei2009@163.com (P.-F.F.); siangli@163.com (S.-A.L.); ly18065176356@163.com (Y.L.); 2 Fujian Zhuyian Agriculture Development Co., Ltd., Fuzhou 350000, China

**Keywords:** *Giardia duodenalis*, prevalence, black goat, Fujian Province

## Abstract

*Giardia duodenalis* is a zoonotic parasite that causes gastrointestinal diseases in both humans and animals. To evaluate the prevalence and genetic diversity of *G. duodenalis* in black goats, we collected 539 fecal samples from nine districts in Fujian Province, China. The presence of *G. duodenalis* was confirmed through nested PCR targeting the SSU rRNA gene, and genotyping was performed at the beta-giardin, glutamate dehydrogenase, and triosephosphate isomerase loci. Among the samples, 115 tested positive, yielding an overall infection rate of 21.34%. Assemblages A and E were identified, with assemblage E being predominant. Statistical analysis revealed significant regional differences in infection rates (*p* < 0.01), with Zhangzhou exhibiting the highest infection rate (39%) and Fuzhou the lowest (3.13%). No significant differences in infection rates were observed based on age: 24.56% (56/228) for goats <1 year, 14.92% (27/181) for goats 1–2 years, 26.8% (26/97) for goats 2–3 years, and 18.18% (6/33) for goats ≥ 3 years. Similarly, no significant differences were found between sexes: 24.84% (40/161) for males and 19.84% (75/378) for females. Notably, assemblage A, a zoonotic genotype, was detected, indicating a potential risk of cross-species transmission. This study contributes to a deeper understanding of *G. duodenalis* in black goats and provides critical data for the development of targeted control strategies in Fujian Province.

## Simple Summary

*Giardia duodenalis* is a parasite that infects the intestines of both animals and humans, leading to health concerns and economic losses in livestock farming. In black goats, infections can cause severe diarrhea or long-term asymptomatic carriers, which impacts animal husbandry. This study aimed to determine the prevalence of *G. duodenalis* in black goats from nine districts of Fujian Province, China. A total of 539 goat fecal samples were collected and analyzed using nested PCR targeting the SSU rRNA gene. Further analyses focused on the beta-giardin, glutamate dehydrogenase, and triosephosphate isomerase genes. Results showed that 115 samples (21.34%) tested positive for *G. duodenalis*, with assemblages A and E detected. Statistical analysis revealed significant differences in infection rates between regions but no significant variation by age or gender. These findings contribute valuable data for understanding *G. duodenalis* in black goats and for improving prevention strategies in livestock.

## 1. Introduction

*Giardia duodenalis* is a flagellated protozoan parasite first identified in human feces in 1681. Mammals become infected by ingesting cysts of *G. duodenalis* through contaminated food or water via the fecal-oral route [1,2]. This parasite is one of the most common causes of diarrhea globally, affecting over 300 million people annually, particularly in developing and least-developed countries. Infected hosts often suffer from gastrointestinal distress, malnutrition, and stunted growth over extended periods. Children and individuals with weakened immune systems are especially vulnerable to *G. duodenalis* infections [3]. The lack of commercial vaccines and specialized treatments for *G. duodenalis* leads to significant economic losses in livestock production [4,5]. Additionally, adult animals that are asymptomatic carriers play a crucial role in the epidemiology of *G. duodenalis*, as they can spread the parasite to other susceptible animals without showing any symptoms [4].

The genus *Giardia* includes six species characterized by *G. duodenalis* morphological features: *G. duodenalis*, *Giardia muris*, *Giardia minor*, *Giardia psittaci*, and *Giardia agilis*. Among these, *G. duodenalis* has eight genetic assemblages (A-H) [6]. Assemblages A and B can infect both humans and animals, and assemblages C through H primarily infect animals.

Assemblages C and D, for example, have been found in domestic dogs and other canines. Assemblage F predominantly infects cats. Assemblage H primarily affects wild animals [7,8,9,10,11,12]. Notably, assemblage E is the most frequently detected in goats worldwide, followed by assemblages A and B [13].

In 1979, the World Health Organization (WHO) listed giardiasis as one of the top ten parasitic diseases threatening human health. The prevalence of giardiasis ranges from 2% to 7% in developed countries but is significantly higher (20–30%) in developing and developing countries [14]. The reported prevalence rates in developed countries include 0.11% in the United States, 4.6% in Canada, 2% in the United Kingdom, and 0.63% in Italy [15,16,17,18]. The reported prevalence rates in developing countries include 23.8% in Brazil, 12.7% in Bangladesh, 23.7% in Malaysia, and 20.3% in Thailand [19,20,21,22].

Epidemiological surveys in China indicate an average infection rate in humans of 0.85%, with a peak infection rate of 9.5% reported in Shanghai [23]. *G. duodenalis* infections have also been widely documented in Chinese cattle, with the first case recorded in Guangdong Province in 2006 [23]. The average infection rate in cattle was 5.43%, with a peak of 18.87% in Shanxi Province [23]. In sheep and goats, the average infection rate is 6.07%. In Henan Province, 24.31% of goats were infected, and the highest infection rate of 27.78% was reported in Chongqing municipality [23]. However, the infection rate in animals from Fujian Province remains unknown.

In order to deepen the understanding of the epidemiology of *G. duodenalis* in Fujian Province, this study utilized molecular detection and genotype identification of *G. duodenalis* in sheep feces from nine administrative regions. There are several methods available for detecting *G. duodenalis*, with fecal microscopy being the most common. In order to provide more accurate detection results, nested PCR was used to analyze the samples and amplify the genes of *G. duodenalis* for genotype identification. These findings offer foundational data for the prevention and control of *G. duodenalis* in black goats in Fujian, and they deepen our comprehension of the genetic makeup and zoonotic potential associated with this parasite.

## 2. Materials and Methods

### 2.1. Study Areas and Sample Collection

**Figure 1:**
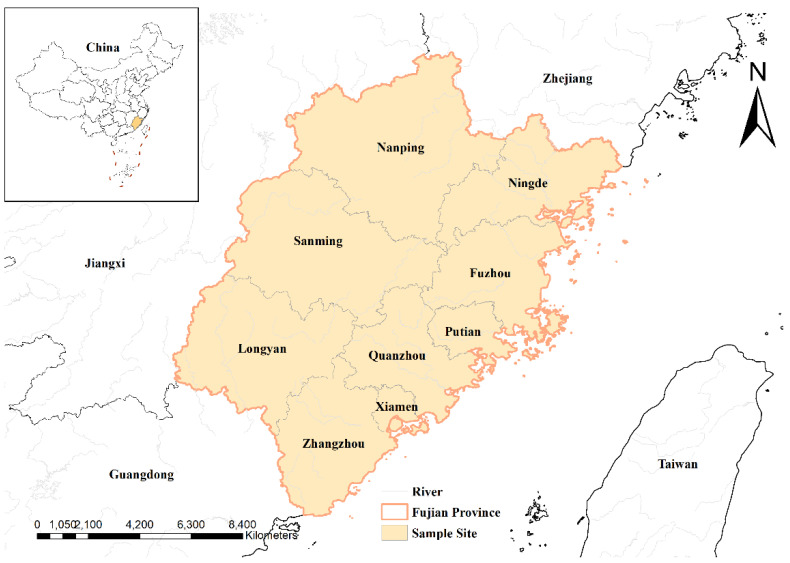
Distribution of sampling sites in Fujian.

Between September and November 2023, a total of 539 fecal samples were randomly collected from black goats on farms in nine administrative districts of Fujian Province (for collection details, refer to Table 1 and Figure 1). The sample set included 161 samples from male goats and 378 from female goats. Age categorization consisted of 228 samples from goats aged ≤1 years, 181 from goats aged 1–2 years, and 97 from goats aged 2–3 years. Fecal samples were obtained directly from the rectum of each goat, individually sealed to prevent cross-contamination, and labeled with information regarding region, gender, and age. The samples were subsequently refrigerated and transported to the laboratory, where they were stored at −80 °C until further analysis.

### 2.2. Genomic DNA Extraction and PCR Amplifications

Total genomic DNA was extracted from approximately 200 mg of each fecal specimen using the Genomic DNA Extraction Kit (TianGen Biotech, Beijing, China). All DNA samples were stored at −20 °C until required for subsequent experiments. The 292bp fragment for the small subunit (*SSU)* rRNA gene by nested PCR amplification was to screen *G. duodenalis*-positive samples. *G. duodenalis*-positive samples were analyzed by MLGs using the three loci in beta-giardin (*bg*), glutamate dehydrogenase (*gdh*), and triosephosphate isomerase (*tpi*). The sizes of the fragments for *bg*, *gdh*, and *tpi* are 511 bp, 530 bp and 530 bp, respectively. PCR reactions were carried out using a thermocycler, with 2 µL of genomic DNA (for primary PCR) or 2 µL of PCR amplification product (for secondary PCR), 2.5 µL of 10 × PCR Buffer, 2 µL of dNTPs, 0.2 µL of Ex Taq polymerase, 0.25 µL of each primer, 1.5 µL of MgCl_2_ (all form Takara, Dalian, China), and ddH_2_O added to a final volume of 25 µL.

### 2.3. Genotype Identification

The identification of *G. duodenalis* was performed using nested PCR based on the amplification of the SSU rRNA gene in black goat fecal samples. These amplification products were stored at 4 °C for short-term use or at −40 °C for long-term storage.

Positive *G. duodenalis* samples were amplified using the *bg*, *gdh*, and *tpi* loci to determine their genotypes. The primers and amplification used in this study have been previously described [3,24]. Amplification products were stored at 4 °C for short-term use or at −40 °C for long-term storage.

### 2.4. Sequencing and Phylogenetic Analysis

Following amplification, the products were analyzed by gel electrophoresis. A 1.0% agarose gel containing nucleic acid dye was prepared. Each sample (5 µL of second-round PCR product mixed with 1 µL of 6× loading buffer) was loaded into the wells, with the Takara DL2000 Marker (Takara, Dalian, China) used as a molecular weight reference. Electrophoresis was performed at 125 V for 30 min in a TAE buffer (Shanghai Toscience Biotechnology Co., Ltd., Shanghai, China). The gel was visualized using a UV gel imaging system, and the results were photographed and saved. Positive PCR products were stored at 4 °C for short-term storage or at −40 °C for long-term storage. All positive secondary PCR products were sent to Sangon Bioengineering Ltd., (Shanghai Sangon Biotechnology Co., Ltd, Shanghai, China) for bidirectional sequencing. Sequence editing and alignment construction were performed using DNAstar 7.1 software, and the final sequences were submitted to the NCBI database for comparative analysis to identify *G. duodenalis* genotypes and polymorphisms. Phylogenetic trees were constructed using the Neighbor-Joining (NJ) method with 1000 bootstrap replicates in Mega11. Separate phylogenetic trees were generated based on identified genotypes or subtypes.

### 2.5. Statistical Analysis

Statistical analysis of the infection rate of *G. duodenalis* in black goats across different districts, age groups, and sex categories was conducted using the *χ^2^* test in SPSS Statistics 26.0. The analysis also incorporates a *t*-test and Bonferroni correction for multiple comparisons. Differences between groups were considered statistically significant when *p* < 0.05.

## 3. Results

### 3.1. The Positivity Rate of G. duodenalis

The SSU rRNA gene was employed for screening positivity to assess the prevalence of *G. duodenalis* in black goats. A total of 539 fecal samples from black goats, collected across nine administrative districts of Fujian Province, were analyzed using nested PCR. The results indicated that 115 samples tested positive, with a positivity rate of 21.34%. Partial results of the amplified products are displayed in the gel electrophoresis.

### 3.2. Prevalence and Genotypic Diversity of G. duodenalis in Black Goats

Infection rates varied significantly across regions, ranging from 3.13% to 39%. The highest prevalence was observed in Zhangzhou City (39%, 32/89), followed by Putian City (37.1%, 23/62) and Longyan City (31.58%, 24/76) (Table 2). Sequence analysis of the *gdh*, *tpi*, and *bg* loci, in conjunction with phylogenetic analysis of the *tpi* locus, revealed that the *G. duodenalis*-positive samples belonged to assemblages E (114) and A (1). Statistical analysis further revealed significant differences in *G. duodenalis* detection rates among the nine districts, emphasizing the importance of considering regional variation when developing control strategies.

### 3.3. Age-Elated Infection Rates of G. duodenalis

The infection rate of *G. duodenalis* in black goats of varying ages across nine administrative districts in Fujian Province is a key factor in understanding the epidemiology of this parasite. The overall infection rates ranged from 0% to 26.8%. Specifically, the detection rates were 24.56% (56/228) for goats aged ≤1 year, 14.92% (27/181) for those aged 1–2 years, 26.8% (26/97) for those aged 2–3 years, and 18.18% (6/33) for goats aged ≥3 years (Table 3). Notably, both assemblages E and A were detected in goats aged 1 year or younger, while assemblage E was found exclusively in goats aged 1–3 years and those older than 3 years. Statistical analysis revealed no significant differences in the detection rates of *G. duodenalis* among the different age groups (*p* > 0.05), suggesting that age may not be a critical factor influencing infection rates in this population.

### 3.4. Prevalence of G. duodenalis Infection in Male and Female Black Goats

The infection rate of *G. duodenalis* in male and female black goats across nine administrative districts in Fujian Province serves as a key indicator of the parasite’s epidemiological patterns. The overall infection rates ranged from 19.84% to 24.84%. Specifically, the infection rate was 24.84% (40/161) for males and 19.84% (75/378) for females (Table 3). This gender distinction is important for understanding the transmission dynamics. Statistical analysis revealed no significant difference in the detection rates of *G. duodenalis* between genders (*p* > 0.05), suggesting that both sexes exhibit similar susceptibility to the infection.

### 3.5. Genotyping of G. duodenalis

**Figure 2:**
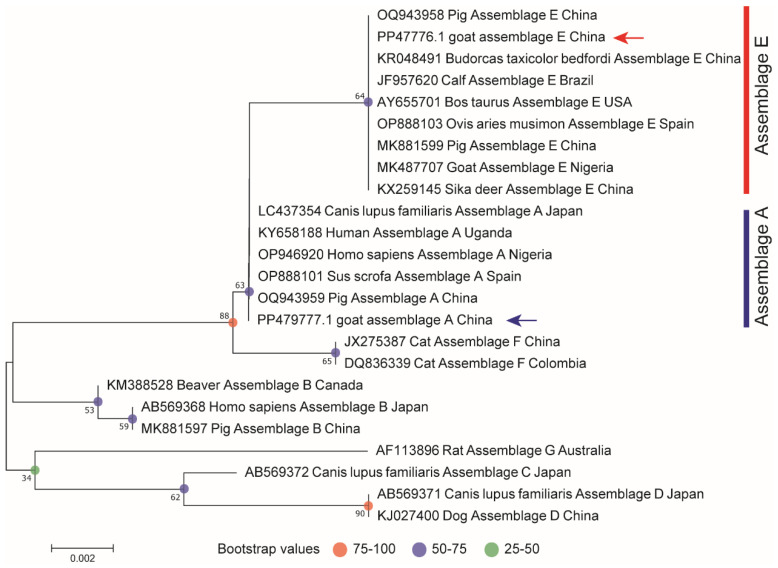
The phylogenetic tree illustrating the evolutionary relationships of *G. duodenalis* from sheep was constructed based on SSU rRNA gene sequences using maximum likelihood analysis. Sequences representative of each sequence type identified in this study were included in the phylogenetic analysis. The arrow indicates the genotypes identified in this experiment.

Two different sequences corresponding to the SSU rRNA were obtained from the 115 positive samples and subsequently submitted to the GenBank database (IDs: PP479776-PP479777). The evolution of the SSU rRNA was analyzed using MEGA 11 software, employing Clustal W for alignment and the maximum likelihood (ML) method for phylogenetic analysis (Figure 2).

### 3.6. G. duodenalis MLST Typing

A total of 115 positive samples were identified based on the SSU rRNA locus. Among these, 35 samples tested positive for the *bg* locus, yielding an amplified fragment of 511 bp, which corresponds to an infection rate of 30.4% (35/115). Likewise, 24 samples were positive for the *gdh* locus, with an amplified fragment length of 530 bp, representing an infection rate of 20.8% (24/115). Additionally, 27 samples tested positive for the triosephosphate isomerase (*tpi)* locus, with a target fragment of 530 bp, resulting in an infection rate of 23.4% (27/115). *bg*, *gdh*, and *tpi* partial results of the amplified products are displayed in the gel electrophoresis.

Genotyping of the 115 positive samples revealed three distinct genotypes: assemblage A, A1 and E. Specifically, one sample was identified as assemblage A, one as assemblage A1, and 84 as assemblage E. At the *bg* locus, one sample of assemblage A and 34 samples of assemblage E were detected, while at the *gdh* locus, 24 samples of assemblage E were identified. At the *tpi* locus, one sample of assemblage A1 and 26 samples of assemblage E were found (Table 4 and Table 5). The sequencing of three loci from 17 samples has been successfully completed. One sample showed mixed infection with combinations A, A1, and E, while the other 16 samples were combinations E. In total, two novel multi-locus genotypes (MLGs) were identified and designated as MLG I and MLG II (Table 6).

**Figure 3:**
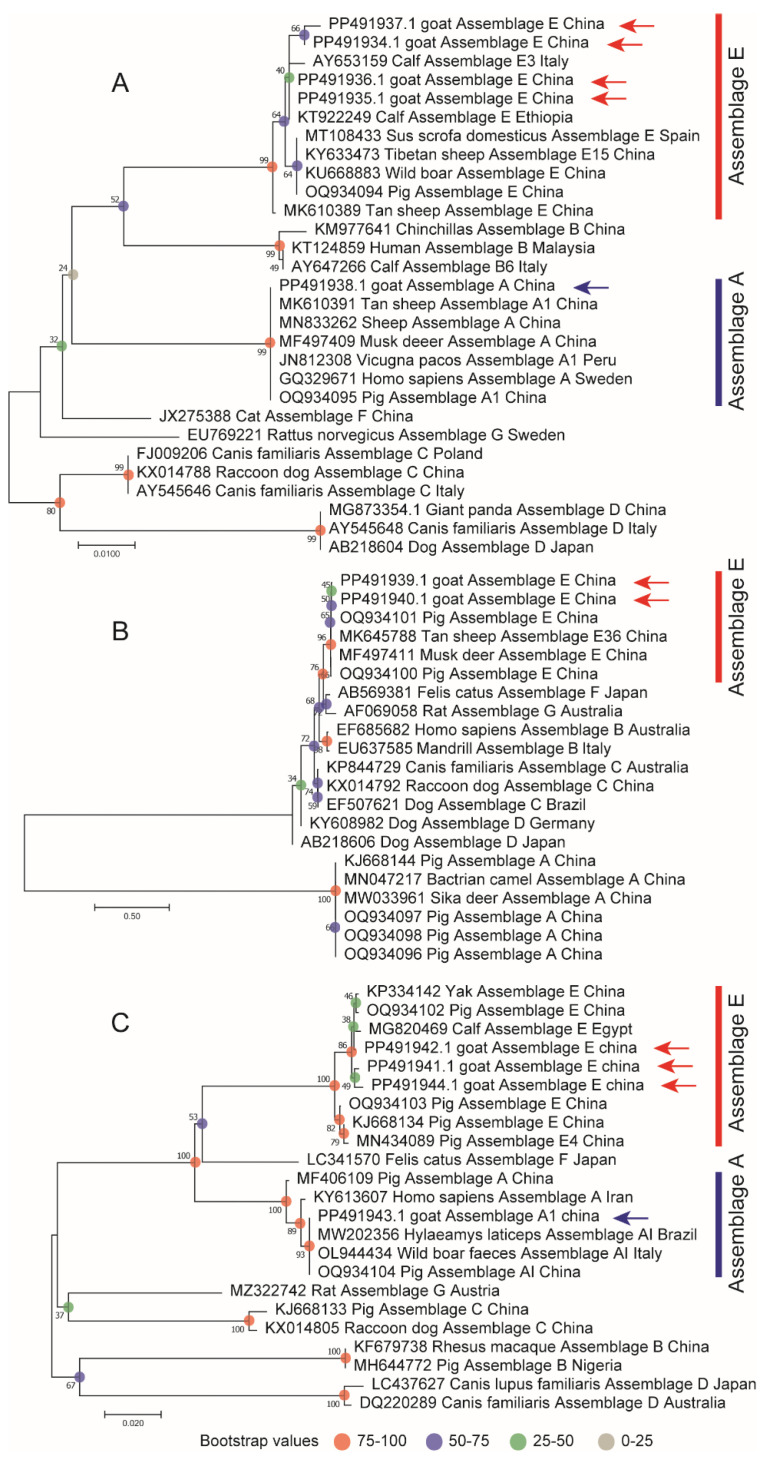
Phylogenetic evolutionary tree diagram of the *bg* (**A**), *gdh* (**B**), and *tpi* (**C**) loci. The arrow indicates the genotypes identified in this experiment.

DNA sequences from the *bg*, *gdh*, and *tpi* loci were subsequently obtained and submitted to the GenBank database. Specifically, five sequences representing the *bg* locus were obtained from the 35 *bg*-positive samples (IDs: PP491934-PP491938), two sequences from the 24 *gdh*-positive samples (IDs: PP491939-PP491940), and four sequences from the 27 *tpi*-positive samples (IDs: PP491941-PP491944). Phylogenetic analysis was performed using Clustal W alignment and the maximum likelihood (ML) method to construct evolutionary trees for the *bg*, *gdh*, and *tpi* loci (Figure 3). The results indicate that the detected assemblage E consistently clusters with the reported assemblage E, showing a close relationship with assemblage E found in both cattle and goats. Notably, the detection of assemblage A in the *bg* genotype and assemblage A1 in the *tpi* genotype aligns with prior studies that suggest assemblage A1 is predominantly found in animals.

## 4. Discussion

*G. duodenalis* infection is relatively common in black sheep, though its prevalence differs greatly, ranging from Anhui (1.62%), Qinghai (2.88%), and Henan (3.4%), Inner Mongolia (28.93%) and Guangdong (24.78%) [25,26,27]. In comparison to other provinces, the notably higher infection rate of *G. duodenalis* in black goats in Fujian Province underscores its widespread prevalence in the region, thereby necessitating further investigation into its epidemiology and molecular characteristics within livestock populations. The detection of two genotypes, assemblages E and A, in the black goats of Fujian Province represents a significant finding. Assemblage E primarily infects livestock, which was identified in 98.26% of the positive samples, particularly ruminants, such as goats and cattle. This genotype’s dominance in livestock is consistent with findings from previous studies across various regions [25,28,29,30]. In contrast, the detection of assemblage A, a common zoonotic genotype, in black goats is noteworthy, as assemblage A infections have been found in a wide range of hosts, including humans, cats, dogs, and wildlife [26]. The presence of this genotype in black goats suggests a potential zoonotic risk to farmers and others in close contact with these animals, highlighting the critical need for preventive measures. Interestingly, this study found no significant differences in infection rates across different age groups of black goats, which contrasts with previous research showing higher susceptibility in younger animals [31,32]. The lack of significant differences in infection rates based on gender and age among black goats may be attributed to various factors, including differences in feeding practices, sample sizes, and dietary components. In black goat breeding facilities in Fujian, the widespread use of fecal leakage boards facilitates the rapid removal of waste. Proactive feces management strategies are critical for controlling the proliferation and survival of parasites. No significant differences in infection rates were observed among different ages, but regional differences in infection rates were observed, with Putian City showing the highest prevalence compared with other regions. These differences may be attributed to various factors, including environmental conditions, local agricultural practices, and the immune status of the animals. The subtropical climate of Fujian, the high humidity, moderate temperatures, and frequent rainfall may further facilitate the persistence and transmission of *G. duodenalis* in this region for extended periods [33]. These environmental factors, rather than host age, are likely to play a significant role in the transmission dynamics of the parasite in this region.

The study also identified two MLG I and MLG II of the *bg*, *gdh*, and *tpi* genes. MLG analysis provides higher resolution than single-locus genotyping, revealing more detailed genetic diversity of *G. duodenalis* strains. This study confirms previous findings that assemblage E is the predominant strain in livestock, with mixed infections of assemblages A and E being rare but present [25,28,29,30]. Assemblage A1 was detected in only one case at the *tpi* locus. While MLG analysis is more sensitive than single-copy markers, it still showed variability in its efficiency, with some loci, such as *bg*, yielding more positive samples than others. This variability highlights the need for comprehensive multi-locus typing in molecular epidemiological studies.

The zoonotic potential of *G. duodenalis* is particularly concerning. While assemblage E is primarily associated with livestock, recent studies have reported an increasing incidence of human infections [34,35,36,37]. The detection of assemblage A, a known zoonotic genotype, in black goats indicates that farmers and other individuals in close contact with these animals may be at risk of infection. The high prevalence of *G. duodenalis* in black goats in this region further emphasizes the need for targeted public health measures to prevent zoonotic transmission.

This study enhances our understanding of the epidemiology and genetic diversity of *G. duodenalis* in black goats in Fujian Province. The identification of both assemblage E and zoonotic assemblage A highlights the need for continued surveillance of this pathogen in livestock populations. Future research should focus on the zoonotic potential of *G. duodenalis* in both humans and animals across China to more accurately assess the risks and develop more effective control strategies.

## 5. Conclusions

This study provides the first molecular evidence of *G. duodenalis* infection in black goats from Fujian Province, China, with an overall infection rate of 21.34% based on the analysis of 539 fecal samples across nine districts. The infection rates varied significantly across regions, with the highest prevalence of 39% recorded in Zhangzhou City. Genotyping revealed that assemblage E, commonly associated with livestock, was the dominant genotype, while assemblage A was detected at a lower frequency, highlighting potential zoonotic risks. The findings showed no significant differences in infection rates based on gender or age, suggesting these factors may not significantly influence susceptibility to *G. duodenalis* infection. These research results enrich our understanding of the epidemiology and genetic diversity of *G. duodenalis* in livestock and emphasize the need for region-specific control measures to prevent the spread of this parasite. Given the zoonotic potential, especially with assemblage A, there is an urgent need for effective strategies to mitigate cross-infections between animals and humans, thereby enhancing overall disease prevention and public health protection.

## Figures and Tables

**Table 1:** Sampling information of Fujian region.

Region	Gender	Age					Summation	Total
		1 Years	2 Years	3 Years	4 Years	5 Years		
Putian	Male	9	0	7	2		62	539
	Female	10	7	8	16	3		
Sanming	Male	6	10	2			73	
	Female	17	33	4	1			
Quanzhou	Male	5	1	1			39	
	Female	10	6	11	5			
Zhang zhou	Male	14	8	1			82	
	Female	13	32	13	1			
Longyan	Male	21	7	2			76	
	Female	19	16	11				
Xiamen	Male	7	3				16	
	Female	1	6					
Nanping	Male	12	11	1			70	
	Female	18	18	9	1			
Ningde	Male	17	2				57	
	Female	27	9	1		1		
Fuzhou	Male	10					64	
	Female	12	15	23	3	1		

**Table 2:** Genotypes of *G. duodenalis* in different areas of Fujian Province.

District	Sample Number	Infection Rate	Assemblages
Sanming	73	10 (13.7%)	E (10)
Putian	62	23 (37.1%)	E (23)
Quanzhou	39	3 (7.69%)	E (3)
Xiamen	16	2 (12.5%)	A (1), E (1)
Zhang zhou	82	32 (39%)	E (32)
Fuzhou	64	2 (3.13%)	E (2)
longyan	76	24 (31.58%)	E (24)
Ningde	57	5 (8.78%)	E (5)
Nanping	70	14 (20%)	E (14)
Total	539	115 (21.34%)	A (1), E (114)

**Table 3:** Prevalence and assemblage distribution of *Giardia duodenalis* by age and gender of black goats.

Type		No. Examined	No. Positive (%)	Genotypes	
				A	E
Gender	Male	161	40 (24.84)	1	39
	Female	378	75 (19.84)	-	75
Age	≤1 year	228	56 (24.56)	1	55
	1–2 year	181	27 (14.92)	-	27
	2–3 year	97	26 (26.8)	-	26
	≥3 year	33	6 (188)	-	6
	Total	539	115 (21.34)	1	114

**Table 4:** Genotypes of three loci in different regions of Fujian Province.

District	Sample Number	Genotype		
		*bg*	*gdh*	*tpi*
Sanming	73	E (8)	E (4)	E (5)
Putian	62	E (7)	E (3)	E (5)
Quanzhou	39	E (2)	E (2)	E (2)
Xiamen	16	A (1), E (1)	E (2)	A1(1), E (1)
Zhang zhou	82	E (2)	E (1)	E (3)
Fuzhou	64			
Longyan	76	E (9)	E (7)	E (6)
Ningde	57	E (3)	E (3)	E (2)
Nanping	70	E (2)	E (2)	E (2)
Total	539	A (1), E (34)	E (24)	A1(1), E (26)

**Table 5:** Genotypic analysis of three genetic loci and assemblage distribution of *Giardia duodenalis* by age and gender of black goats.

Type		No. Examined	Genetic Loci					
			*bg*		*gdh*		*tpi*	
			A	E	A	E	A1	E
Gender	Male	161	1	16	-	12	1	12
	Female	378	-	18	-	12	-	14
Age	≤1 year	228	1	24	-	17	1	19
	1–2 year	181	-	6	-	6	-	5
	2–3 year	97	-	4	-	1	-	2
	≥3 year	33	-	-	-	-	-	-
	Total	539	1	34	-	24	1	26

**Table 6:** MLST typing of *G. duodenalis* of Black sheep.

Regions	Sample Number	Genotype			MLGs
		*bg*	*gdh*	*tpi*	
Longyan	Ly (1, 32, 35, 36, 38, 52)	E	E	E	I
Nanping	Np (46, 52, 51, 57)	E	E	E	I
Putian	Pt (28)	E	E	E	I
Quanzhou	Qz (34, 36)	E	E	E	I
Sanming	Sm (44)	E	E	E	I
Zhangzhou	Zz (72)	E	E	E	I
Xiamen	Xm (14)	E	E	E	I
	Xm (4)	A	E	A1	II

## Data Availability

The original contributions presented in this study are included in the article.
